# The Epidemiology of Primary Central Nervous System Tumors at the National Neurologic Institute in Saudi Arabia: A Ten-Year Single-Institution Study

**DOI:** 10.1155/2020/1429615

**Published:** 2020-02-15

**Authors:** Amna Almutrafi, Yara Bashawry, Wafaa AlShakweer, Musa Al-Harbi, Abdullah Altwairgi, Sadeq Al-Dandan

**Affiliations:** ^1^Department of Anatomical Pathology, King Fahad Medical City, Riyadh, Saudi Arabia; ^2^Department of Pathology, King Abdulaziz Medical City, Riyadh, Saudi Arabia; ^3^Research Center, King Fahad Medical City, Riyadh, Saudi Arabia; ^4^Department of Pediatric Hemato-Oncology, Comprehensive Cancer Center, King Fahad Medical City, Riyadh, Saudi Arabia; ^5^Department of Medical Oncology, Comprehensive Cancer Center, King Fahad Medical City, Riyadh, Saudi Arabia; ^6^Department of Pathology, Maternity and Children Hospital Al-Ahsa, Al-Ahsa, Saudi Arabia

## Abstract

**Objectives:**

This study is aimed at describing the epidemiological trends of primary CNS tumors in children and adults at the National Neurologic Institute in Saudi Arabia.

**Methods:**

A retrospective epidemiological approach was used where data was obtained from the department of pathology registry files and pathology reports. The records of all patients registered from January 2005 to December 2014 with a diagnosis of primary CNS tumor (brain and spinal cord) were selected. Data about sex, age, tumor location, and histologic type were collected. The classification was based on the International Classification of Diseases for Oncology, 3rd Edition (ICD-O-3).

**Results:**

Nine hundred and ninety-two (992) cases of primary CNS tumors throughout the ten years (2005 to 2014) were reviewed. There were 714 (71.97%) adults and 278 (28.02%) in the pediatric age group. Nonmalignant tumors dominated the adult population (60.08%) while malignant tumors were more frequent in the pediatric population. Gliomas constituted the most common neoplastic category in children and adults. The most common single tumor entity was meningioma (26.99%, ICD-O-3 histology codes 9530/0, 9539/1, and 9530/3). Medulloblastomas (ICD-O-3 histology codes 9470, 9471, and 9474) were the most common single tumor entity in the pediatric age group (26.62%).

**Conclusions:**

This is an institution-based, detailed, and descriptive epidemiological study of patients with primary CNS tumors in Saudi Arabia. In contrast to other regional and international studies, the medulloblastomas in our institution are more frequent than pilocytic astrocytomas. Limitations to our study included the referral bias and histology-based methodology.

## 1. Introduction

The worldwide incidence age-standardized rates (ASR) of brain and nervous system cancer in high/very-high HDI (Human Development Index) regions versus low/medium HDI regions was 5.0 and 2.4 for men and 4.0 and 1.7 for women (Saudi Arabia is classified as very-high HDI according to the United Nations Development Program 4-tier system), respectively. These incidence rates were approximately twofold higher in high/very-high HDI countries compared with low/medium HDI countries and slightly higher for males compared to females [1].

Even though about half of these tumors are benign, they may cause substantial morbidity. Brain tumors are the leading cause of cancer death in children and the third cause of death related to cancer in adolescents and adults [2]. In the Gulf Cooperation Council (GCC) countries, brain cancer is the tenth most common cancer (1998-2007) [2]. The overall age-standardized rate (ASR) of brain cancer between1998 and 2007 was 2.4 for males and 1.6 for females per 100,000 populations [2]. The incidence ASR for brain and CNS cancer in Saudi Arabia in 2008-2012 was 2.2 for males and 1.5 for females (per 100,000 populations). Brain and CNS cancers comprised 3.70% of all cancers in males and 2.30% of all cancers in females [3].

In Saudi Arabia, ASR in 2014 was 2.2 for males and 1.7 for females per 100,000 populations [4]. According to the Saudi Cancer Registry, there were 329 cases of brain cancer, accounting for 2.8% of all reported cancer cases in 2014. Brain cancer is ranked 11^th^ among males and 10^th^ among females in 2014 [4]. According to the GLOBOCAN 2018, there were 569 cases of brain and nervous system cancers, accounting for 2.3% of all reported cancer cases in 2018 and ranking 14^th^ among all cancer cases [5].

The National Neuroscience Institute (NNI) at King Fahad Medical City (KFMC) is dedicated to the provision of comprehensive medical care to patients with neurologic diseases in Saudi Arabia. The practice of neurooncology lies at the heart of the NNI objective, and the team of health care providers amalgamate clinical experience with modern technologies to offer the best care to patients. Over the last ten years, the capacity of NNI had progressively increased and so did the number of patients treated for CNS tumors. The rates, trends, and epidemiology of primary CNS tumors at the NNI remain mostly unknown. To understand the current epidemiology of primary CNS tumors in Saudi Arabia, reports that describe the disease according to international reporting standards are needed. There are only a few to define an institution-based frequency or incidence rate of primary CNS in different Saudi Arabian regions [6–9].

Thus, the objective of this study is to outline the epidemiology of primary CNS tumors at King Fahad Medical City, over a ten-year period (2005-2014). Our work will enable us to observe any unusual trend in primary CNS tumor epidemiology and compare our results with local and regional data.

## 2. Materials and Methods

This is a retrospective study carried out using the laboratory information system (CorTTeX), departmental diagnostic registry, and pathology reports at King Fahad Medical City (KFMC), Riyadh, Saudi Arabia. Institutional review board approval was granted before the start of the study. The study evaluated the distribution of primary CNS tumors (brain and spinal cord) for ten years from 2005 to 2014 (KFMC opened in 2004 and the study start date was 2005).

Inclusion criteria included a histopathologic diagnosis of primary brain tumor of any age and sex, availability of clinical data, and histologic slides for confirmation of diagnosis. Exclusion criteria included absence of histologic slides and insufficient clinical data. Nonneoplastic brain lesions, secondary brain tumors (metastases), and scalp and primary bone tumors with intracranial extension were excluded.

Diagnosis and grading of tumors were established according to the 2016 WHO Classification of Tumors of the CNS. Review of the histologic slides was carried out whenever there was an ambiguity in the diagnosis or tumor grade in the pathology reports. Tumors were divided into nonmalignant (WHO grades I and II) and malignant (WHO grades III and IV) categories. The ICD-O-3 coding system was used. The epidemiology profile of the tumors, including the anatomical location, histologic diagnosis, and World Health Organization (WHO) grade in both adult (>18 years) and pediatric populations (0-18 years), was collected. The pediatric population was further divided into infants (0-<1 year), children (1-14 years), and adolescents (15-18). Moreover, age and gender were also recorded.

All statistical analyses, including counts, means, rates, ratios, and proportions, were performed using the SPSS 22.0 software package (SPSS Inc., Chicago, IL, USA). The proportions of malignant to nonmalignant tumors and supratentorial to infratentorial tumors were calculated.

## 3. Result

**Figure 1:**
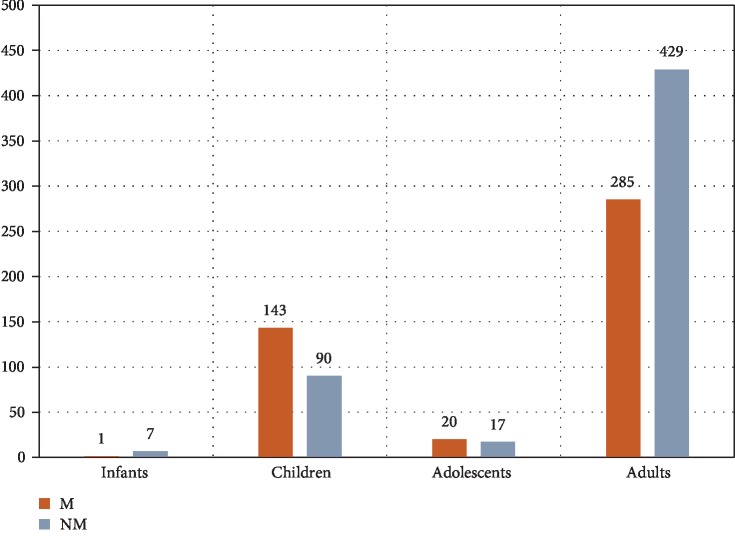
Distribution and proportions of malignant and nonmalignant primary CNS tumors in different age groups.

**Figure 2:**
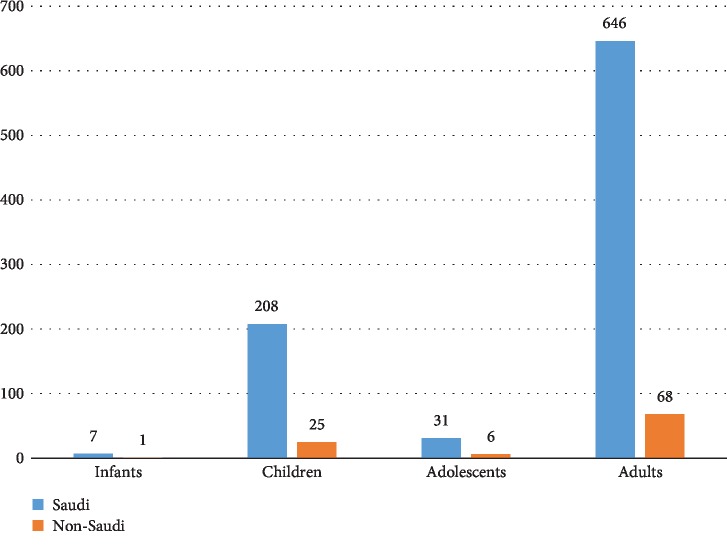
Distribution and proportions of Saudi citizens and non-Saudi residents with primary CNS tumors in different age groups.

**Figure 3:**
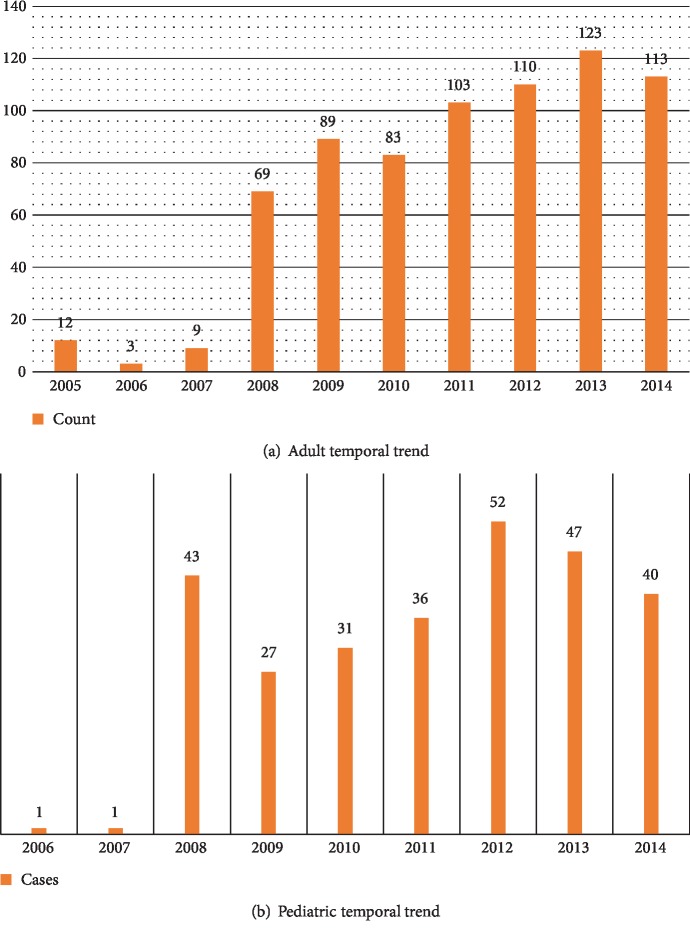
Annual distribution and trend of adult and pediatric primary CNS tumors (2005-2014).

Nine hundred and ninety-two cases of primary CNS tumors were reviewed. There were 714 (71.97%) adults and 278 (28.02%) in the pediatric age group. Nonmalignant tumors dominated the adult population (60.08%), while malignant tumors were more frequent (61.37%) in the pediatric population (Figure 1). There were 892 Saudi citizens and 100 non-Saudi residents distributed among all age groups (Figure 2). The trends of primary CNS tumors over the ten-year period were rising. Years 2012 and 2013 had the highest numbers of brain tumors for pediatric as well as for adult patients (Figures 3(a) and 3(b)).

### 3.1. Adult Population

**Figure 4:**
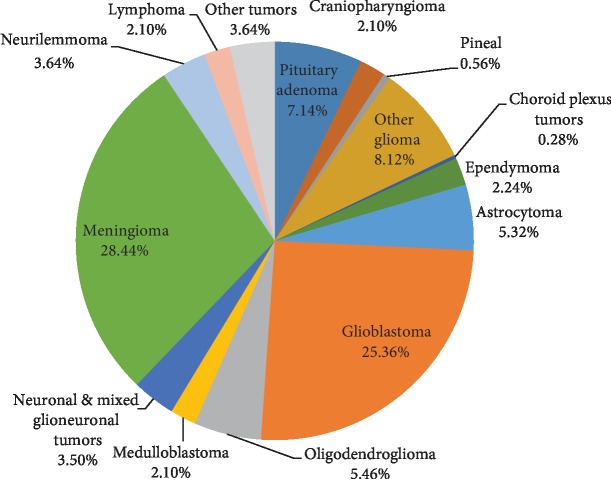
Distribution of all primary CNS tumors by histology diagnosis for adults (>18 years).

The mean age at the time of diagnosis was 45.64 years, with a standard deviation of 15.51 years. There were 373 (52.24%) males. There were 285 (39.92%) malignant tumors. The most common single tumor entity was meningioma (26.99%, ICD-O-3 histology codes 9530/0, 9539/1, and 9530/3) followed by glioblastoma (25.10%, ICD-O-3 histology codes 9440/3 and 9442/3) (Figure 4). Among all meningiomas, 82.75% were WHO grade I, 16.7% were grade II, and 0.49% were grade III.

Nonmalignant tumors included WHO grade I meningiomas (23.5%), pituitary adenoma (7.14%), neurilemmomas (3.6%), neuronal and mixed glioneuronal tumors (3.5%), craniopharyngiomas (2.1%), and low-grade gliomas (WHO grades I and II).

**Figure 5:**
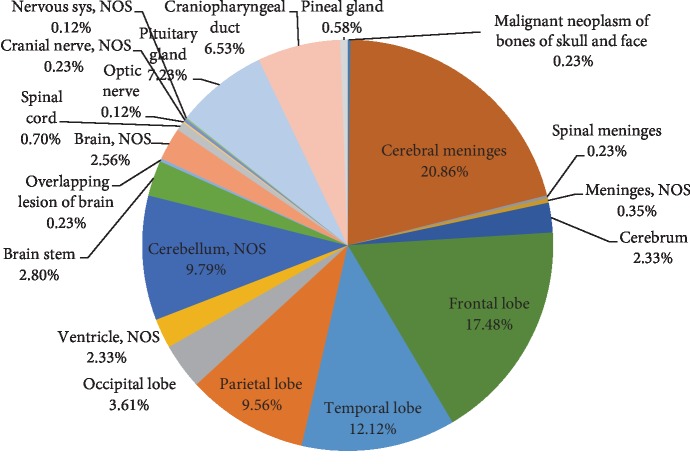
Distribution of all primary CNS tumors by anatomic site for adults (>18 years).

**Figure 6:**
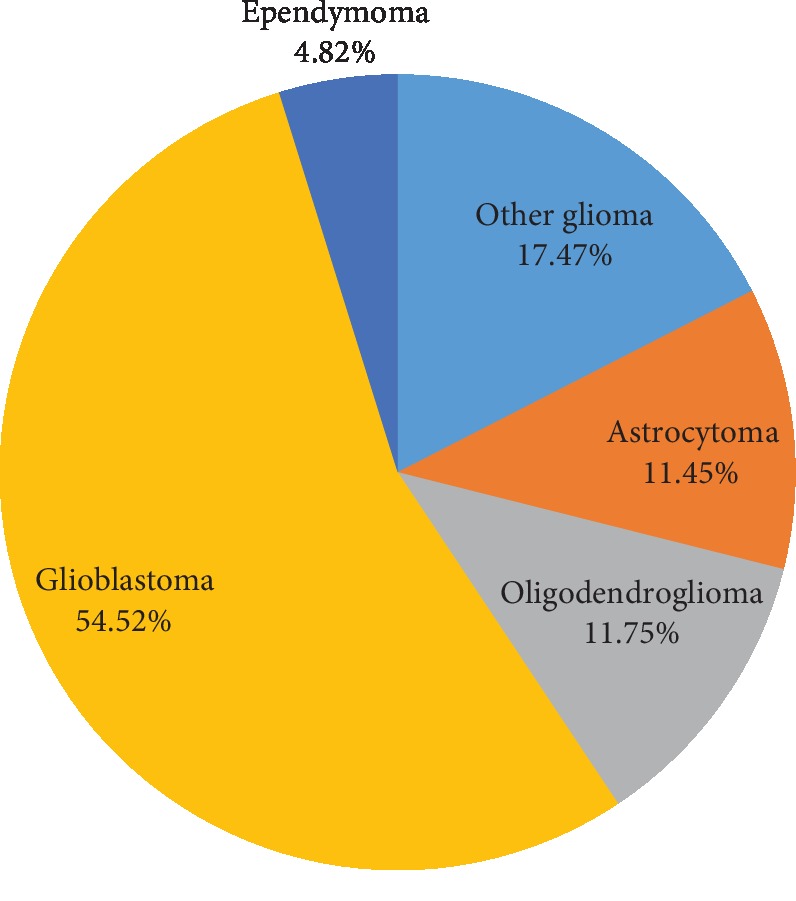
Distribution of all primary CNS gliomas by histologic subtype for adults (>18 years).

Adult tumors were more frequently distributed in the supratentorial compartments, with cerebral meninges (ICD-O-3 site code C70.0) being the most common location followed by intraparenchymal frontal lobe (ICD-O-3 site code C71.1) (Figure 5). Adult gliomas (glioblastomas, astrocytomas, oligodendrogliomas, and ependymomas) were the most frequent (46.49%) neoplastic category, and glioblastoma was the commonest of all (54.52%) (Figure 6).

### 3.2. Pediatric Population

**Figure 7:**
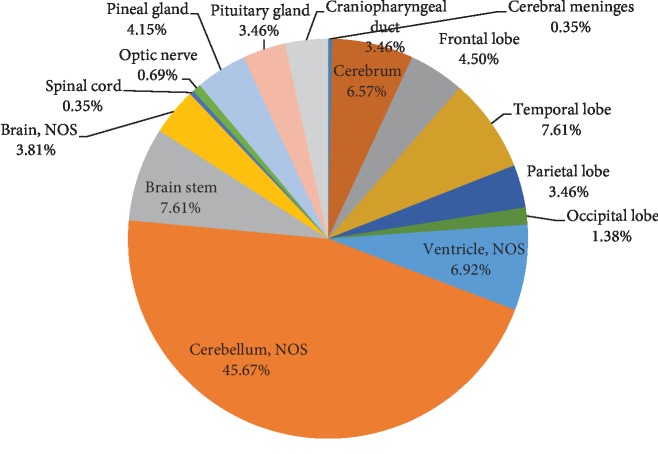
Distribution of all primary CNS tumors by anatomic site for children and adolescents (0-18 years).

The mean age of patients was 7.4 years, with a standard deviation of 4.90 years. There was a male predominance with 167 (60.07%). Most of the tumors were found in the infratentorial location (154/278, 55.39%), including the cerebellum (132 tumors, 45.67%) and brainstem (22 tumors, 7.61%) (Figure 7).

**Figure 8:**
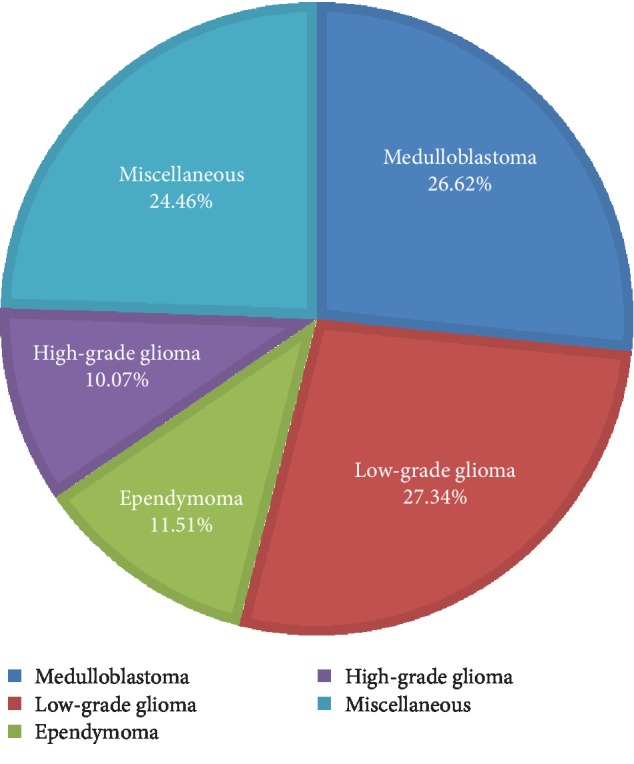
Distribution of all primary CNS tumors by broad histologic category for children and adolescents (0-18 years).

Medulloblastomas (ICD-O-3 histology codes 9470, 9471, and 9474) were the most common single tumor entity in the pediatric age group (26.62%) followed by pilocytic astrocytomas (17.99%, ICD-O-3 histology codes 9421/1), ependymomas (11.51%, ICD-O-3 histology codes 9392/3, 9391/3, and 9394/1), and high-grade gliomas (10.07%) (Figure 8).

Other miscellaneous pediatric tumors constituted 24.46%, and these included atypical teratoid/rhabdoid tumors (2.88%, ICD-O-3 histology code 9508/3), choroid plexus neoplasms (3.60%, ICD-O-3 histology codes 9390/1 and 9390/3), primitive neuroectodermal tumors (2.88%, ICD-O-3 histology code 9473/3), germ cell tumors (2.52%, ICD-O-3 histology codes 9064/3, 9080/0, and 9071/3), craniopharyngiomas (3.24%, ICD-O-3 histology codes 9350/1 and 9351/1), pineoblastomas (2.52%, ICD-O-3 histology code 9362/3), meningiomas (1.08%, ICD-O-3 histology codes 9530/1 and 9538/1), and neuronal and glioneuronal tumors (3.60%, ICD-O-3 histology codes 9413/0, 9505/1, and 9506/1).

**Figure 9:**
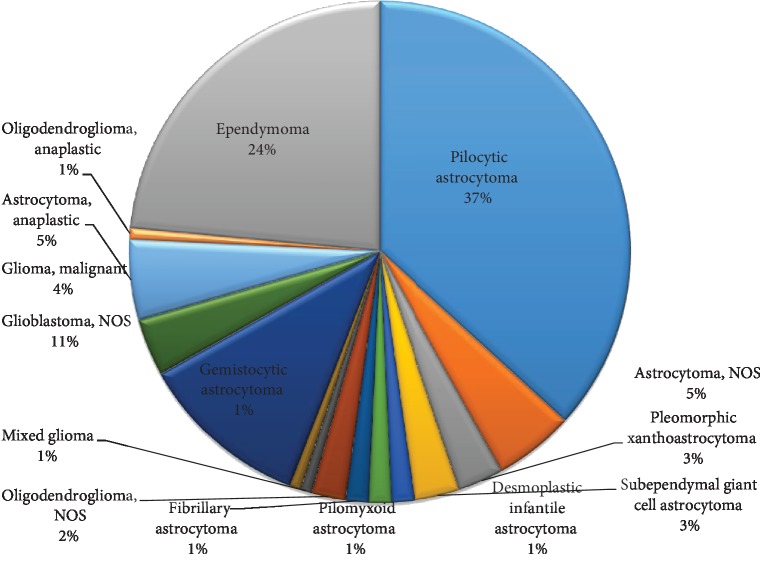
Distribution of all primary CNS gliomas by histologic subtype for children and adolescents (0-18 years).

Similar to the adult group, gliomas constituted the most common (48.93%) neoplastic category, and they included low-grade gliomas, ependymomas, and high-grade gliomas. Pilocytic astrocytomas (36.76%, ICD-O-3 histology code 9421/1) were the predominant glioma tumor for pediatric populations followed by ependymomas (23.53%, ICD-O-3 histology codes 9392/3, 9391/3, and 9394/1) and high-grade gliomas (20.58%, ICD-O-3 histology codes 9440/3, 9380/3, 9401/3, and 9451/3) (Figure 9).

## 4. Discussion

The rising trends of primary CNS tumors over the ten-year period reflect the expansion in the capacity of the National Neurologic Institute and King Fahad Medical City. The reduction of number of tumors in 2014 could be attributable to cancellations of neurosurgical procedures due to the unavailability of Intensive Care Unit (ICU) beds. This was largely caused by the influx of critical patients infected in the outbreaks of the Middle East Respiratory Syndrome Coronavirus (MERS-Cov).

Our results showed that gliomas and especially astrocytomas were the most common pathologic categorical entity similar to a global study conducted by Leece et al. [10]. The most common single tumor entity in adults was meningioma. These findings were similar to other studies in different regions of the Kingdom of Saudi Arabia such as Abha city (meningioma WHO grade I 41.7%) [9], the Western province (meningioma 17.8%) [11], and the Eastern province [12]. Our findings are also similar to other countries in the Middle East, such as Jordan (meningioma 26.2%) and Iran (meningioma 27.8%) [13, 14]. Meningioma was also the leading histologic tumor type worldwide. In the United States, it was 36.1%; France, 30.9%; and Korea, 31.1% [13]. Taha et al. found glioblastoma to be the most common pathological type (32%), but their study was based on neuroepithelial tumors and excluded meningeal-based tumors [15].

Several studies reported the frontal lobe to be the most common site for primary brain tumors in adults [9, 11]. In our study, we found that the cerebral meninges are the most common site followed by the frontal lobe. This could be attributed to the predominance of meningioma over other neoplasms. Our findings are similar to the Center of Brain Tumor Registry of the United States (CBTRUS) where the most common tumor site in adults was the meninges, representing 36.1% [16, 17].

Medulloblastomas were the most commonly reported histology type in the pediatric age group followed by low-grade gliomas with a predominance of pilocytic astrocytoma. Similar to our finding, medulloblastoma was the most common morphological type reported nationally among children by the Saudi Cancer Registry [4]. Previous two studies carried out at King Abdulaziz University Hospital (KAUH) revealed that astrocytoma was more prevalent in the Western region [6, 11]. In the Egyptian pediatric population, the most common intracranial tumors were astrocytomas (35%) followed by medulloblastomas (18.8%). Pilocytic astrocytomas constituted 55% of all astrocytomas and 19.3% of all brain tumors, only slightly ahead of medulloblastomas [18]. The most common tumor found in a Syrian childhood population was medulloblastoma (27.5%), followed by astrocytoma (25.8%) [19]. The lack of ICD-O-3 histology coding in most of these previous works could have resulted in the discrepancy between different studies in different populations.

Additionally, the 2015 CBTRUS report for infant and childhood primary brain and CNS tumors showed pilocytic astrocytoma to be the leading histological type [16]. The Saudi Cancer Registry used a malignancy-based statistical approach where pilocytic astrocytomas (WHO grade I) were omitted, which explains the higher frequency of medulloblastoma in their registry. Our institution had relatively lower rates of glial neoplasms in general (42.97%) and particularly pilocytic astrocytomas (15.3%). Many of the pilocytic astrocytomas were likely decompressed or excised in their original secondary hospitals while most of the medulloblastomas require gross total resection and referral to a tertiary center such as the NNI. The high-grade gliomas are likely underrepresented in our study since the methodology is pathology-driven and most high-grade gliomas are not biopsied (diffuse intrinsic pontine glioma). The referral bias and the pathology-driven methodology could have contributed, in whole or in part, to the relatively higher rates of medulloblastoma in our study.

Among all pediatric gliomas, 42.64% were WHO grade I, 13.23% were WHO grade II, 23.52% were WHO grade III, 11.02 were WHO grade IV, and 9.55% were not graded. Qaddoumi et al. reported similar results in their review of 6212 cases of pediatric glioma (33.7% WHO grade I, 21.8% WHO grade II, 7.9% WHO grade III, 9.0% were WHO grade IV, and 28.5% were unknown). We had lower percentages of tumors with unknown grades (9.55%) compared to the Qaddoumi et al. review (28.5%) because our study is pathology-driven and the histologic slides were reviewed whenever the pathology reports lacked WHO grades [20].

Percentages for pediatric tumors were highest in infratentorial sites (55.39%). These findings are following those of several studies that stated how the infratentorial compartment was reported to be the most common site of brain tumors in the pediatric age group [18, 19].

A systematic review by Khan et al. showed that inadequate reporting of CNS tumor subtypes was observed in registries of developing countries [21]. They suggested establishing a unified reporting system to help improve health management for CNS tumors [16]. Unstandardized histology groupings and reporting can lead to different interpretations and incomparable results between different populations. Chan et al. reviewed the challenges and opportunities of creating cancer registries in developing nations and suggested investing in quality hospital-based and population-based cancer registries with dedicated financial resources and manpower (health care professionals and information technologists) [22].

This study contains the largest institution-based ICD-O and WHO-classified epidemiological analysis of malignant and nonmalignant primary brain tumors in Saudi Arabia in adult and pediatric groups. Our study is limited by the referral bias and histology-based methodology with the exclusion of radiologically diagnosed tumors. This may potentially lead to underestimating the incidence of some gliomas such as diffuse intrinsic pontine gliomas and optic nerve gliomas. The findings in this study are generalizable to other tertiary centers in Saudi Arabia but not to community hospitals.

Most of the reviewed studies of brain tumors in Saudi Arabia are institution-based. The Saudi Cancer Registry reports malignant brain tumors only. We suggest the establishment of a National Brain Tumor Registry in Saudi Arabia similar to the Central Brain Tumor Registry of the United States (CBTRUS). This will help in standardization of diagnostic nomenclature (ICD-O coding) of brain tumors and accurate description of their incidence and survival trends.

## 5. Conclusion

Glioma as a broad diagnostic category was most common in both adult and pediatric age groups. With regard to a single tumor entity, meningioma was the most common primary brain tumor in adults while in the pediatric age group, medulloblastoma was the leading histology.

## Data Availability

The data used to support the findings of this study are restricted by the Institutional Review Board of King Fahad Medical City in order to protect patient privacy. Data are available from Dr. Wafaa Al-Shakweer (Department of Pathology, King Fahad Medical City) for researchers who meet the criteria for access to confidential data.
